# In Vivo Study on Analgesic, Muscle-Relaxant, Sedative Activity of Extracts of* Hypochaeris radicata* and In Silico* Evaluation* of Certain Compounds Present in This Species

**DOI:** 10.1155/2018/3868070

**Published:** 2018-05-13

**Authors:** Tareq Abu-Izneid, Abdur Rauf, Syed Uzair Ali Shah, Abdul Wadood, Mohamed I. S. Abdelhady, Priymenko Nathalie, Domange Céline, Nashwa Mansour, Seema Patel

**Affiliations:** ^1^Faculty of Pharmacy, Umm Al-Qura University, Makkah, Saudi Arabia; ^2^Department of Chemistry, University of Swabi, Anbar, Khyber Pakhtunkhwa 23561, Pakistan; ^3^Department of Pharmacy, University of Swabi, Anbar, Khyber Pakhtunkhwa 23561, Pakistan; ^4^Department of Pharmacy, Abdul Wali Khan University Mardan, Mardan 23200, Pakistan; ^5^Department of Pharmacognosy, Faculty of Pharmacy, Helwan University, Cairo 11795, Egypt; ^6^Toxalim, Université de Toulouse, INRA, ENVT, Toulouse, France; ^7^UMR Modélisation Systémique Appliquée aux Ruminants, AgroPArisTech, Université Paris-Saclay, 75005 Paris, France; ^8^Department of Medicinal Chemistry, Faculty of Pharmacy, Beni-Suef University, Beni-Suef 62111, Egypt; ^9^Bioinformatics and Medical Informatics Research Center, San Diego State University, San Diego, CA 92182, USA

## Abstract

**Background:**

* Hypochaeris radicata *(flatweed) from the family Asteraceae is a medicinal plant found in Europe, Middle East, and India. In folkloric medication, it is used to heal jaundice, dyspepsia, constipation, rheumatism, and hypoglycemia as well as renal problems. Leaves and roots of the plant have antioxidant and antibacterial properties. The plant is a rich source of pharmacologically active phytochemicals; however, it is explored scantily. The objective of the current study was to identify the chemical composition and investigate the* in vivo* biological potency of crude extracts of this plant.

**Methods:**

The crude extract and the fractions were screened for various phytochemical groups of constituents following standard procedures. The acute toxicity was assayed for safe range of dose determination. The analgesic potential of the extract and fractions was assessed by acetic acid-induced writhing test. The muscle-relaxant activity was examined by standard inclined-plane test and traction test. Sedative potential of extract/fractions was assessed by using standard white wood procedures. Furthermore, docking analysis of two compounds present in the ethyl acetate fraction of the plant was assessed against 3D cyclooxygenase-1 and -2 (COX-1 and COX-2).

**Results:**

The extract/fractions of* H. radicata *showed significant analgesic effect in* in vivo* model of peripheral algesia. The docking analysis of previously isolated molecules from the plant also exhibited promising interaction with COX-1 and COX-2. Also, the plant has a mild sedative and muscle-relaxant potential. Thus, our study provided pharmacological rationale for the traditional uses of the plant as analgesic and anti-inflammatory remedy.

**Conclusion:**

The crude extracts and fractions exhibited excellent activity due to active phytochemicals. These active phytochemicals also exhibited promising interaction with COX-1 and COX-2. These findings directed researcher to isolate active compounds from* H. radicata *which may be used as a potential source of active secondary metabolites.

## 1. Introduction

*Hypochaeris radicata*, commonly known as cat's-ear or flatweed, belongs to the family Asteraceae. Its geographical range includes Europe and Indian peninsula (the high hills of Nilgiris and the Western Ghats). Its roots and leaves have been used in traditional medicine as antioxidant, antidiuretic, anti-inflammatory, anticancer, antibacterial, hepatoprotective, and renoprotective [1, 2]. In folkloric system it is used to cure jaundice, dyspepsia, constipation, rheumatism, and hypoglycemia [1].

**Figure 1:**
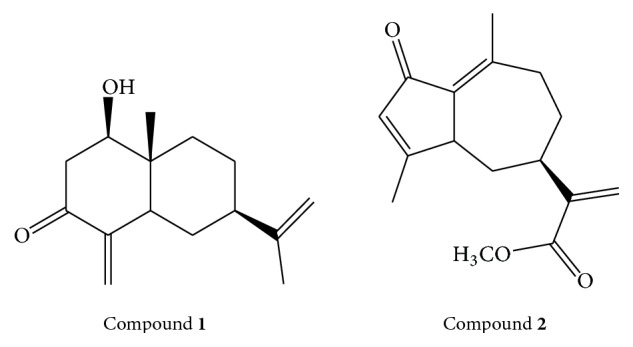
The nonvolatile compounds (**1** and** 2**) present in* H. radicata.*

*H. radicata *is a rich source of pharmacologically active phytochemicals; however, it is evaluated only briefly. Various phytochemicals such as sesquiterpenes, glycosides, germacrene, eudesmane, guaianolides, and phenylbutanoid glycoside have been documented in the existing literature [3]. Previously, some nonvolatile compounds have been isolated from this plant, including (4R,4aR,7R)-7-ethyl-4-hydroxy-4a-methyl-1-methylene-octahydronaphthalen-2(1H)-one (compound** 1**) and methyl 2-((5R,E)-3,8-dimethyl-1-oxo-1,3a,4,5,6,7-hexahydroazulen-5-yl)acrylate (compound** 2**) (Figure 1). These phytochemicals might be responsible for the anti-inflammatory action of this plant. Therefore, we conducted docking analysis for two compounds present in* Hypochaeris radicata*, against cyclooxygenases (COX-1 and COX-2) [1]. In the present study, we have focused on the confirmation and analysis of folkloric anti-inflammatory usage of this Compositae family plant.

## 2. Experimental

### 2.1. Plant Collection

The plant material was collected from Toulouse, France; the plant specimen was identified by well-known plant taxonomist; and then a voucher specimen number, PHG130, was deposited in the herbarium of the Pharmacognosy Department, Faculty of Pharmacy, Umm Al-Qura University, KSA.

### 2.2. Extraction and Fractionation

The sample plants were dried in shade, powdered on drying, and then subjected to cold extraction, using 80% methanol. The extraction was performed thrice to extract the maximum amount crude metabolites. The obtained extract was concentrated using a rotary evaporator which produced 145 gm of crude residue. The crude extract was suspended in minimum amount of distilled water and successively fractionated with various solvents to obtain fractions: hexane (30 gm), chloroform (53 gm), and ethyl acetate (45 gm) as per our reported methods [4]. These obtained fractions were stored in refrigerator for further biological screening.

### 2.3. Phytochemical Analysis

The extract and its isolated fractions were assessed for phytochemical screening before bulk extraction to identify tentative active phytochemicals as per standard procedure [4, 5].

To identify alkaloids in crude extract/fractions, about 0.5 gram sample was warmed with H_2_SO_4_ (2%) and the Dragendorff's reagents were added to each sample. The appearance of orange red precipitate indicated the presence of alkaloids moiety in extract. For tannins detection 0.5 gm sample (extract/fraction) was dissolved in distilled water, heated, and the filtered to remove dissolved impurities. Few drops of ferric chloride were added to each filtrate; the appearance of dark green color in the solution exhibited the presence of tannins. The identification of anthraquinones was achieved by standard procedure. About 0.5 gm of extract and its isolated fractions was boiled with 10% HCl for few minutes on water bath. The mixtures were cooled and then filtered to remove the suspended plant materials. Few drops of ammonia (10%) were added to the mixture and then heated on water bath. The appearance of rose pink color exhibited the presence of anthraquinones moiety. Glycosides were detected by adding 0.5 gm of extract/fractions with hydrochloric acid (hydrolyzed) and the neutralization was achieved with NaOH. Then few drops of Fehling's solution A and then B were added to reaction mixture. The appearance of red color precipitate showed the presence of glycosides in extract/fractions. Reducing sugars were also identified by using reported procedure. The 0.5 g crude extract was shaken with distilled water followed by filtration. After addition of a few drops of Fehling's solution A and B to the filtrates, the mixtures were boiled for few min. Appearance of orange red precipitates showed the presence of reducing sugars. Saponins was identified by mixing each extract and its fractions (0.5 gm) with distilled water and then boiling them. The formation of froth in mixture confirmed the presence of saponins. Flavonoids were identified by using standard protocol. The 0.5 g crude extracts were dissolved in diluted NaOH and few drops of HCl were added. The yellowish solution turned colorless after the addition of HCl, which showed the presence of flavonoids in the extracts. The phlobatannins was detected by mixing 0.5 gm extracts/fractions in distilled water and the filtering them. The filtrates were boiled with 2% HCl solution. The presence of red precipitate indicated the presence of phlobatannins. Steroids were identified by standard procedure. About 0.5 g of the crude obtained extract/fractions was taken in test tube and few drops of acetic acid were added. The mixture was gently heated using burner and then cooled. The mixture was then treated by addition of H_2_SO_4_ dropwise, and the color changed to green indicating the presence of steroids. Terpenoids was detected by using reported methods. About 0.2 g of the extract/fractions was treated with 2 mL CHCl_3_ and then filtered. The filtrates were treated with 3 mL H_2_SO_4_, shaken, and allowed to stand for few minutes. The presence of golden yellow color designates the occurrence of triterpenes. Coumarine was also detected by using reported methods. About 1 mL aqueous extract/fractions was treated with 3 mL of 10% NaOH. The presence of coumarin was detected by the formation of yellow color. Emodin was also detected by using reported methods. About 0.3 gm of the extract/fractions was mixed with 2 mL of NH_4_OH followed by the addition of 3 ml of benzene. A red color formation proved the presence of emodin. Anthocyanin and betacyanins were identified by standard procedure. 0.2 g of plant extract/fractions and 1 mL of NaOH were mixed and heated at 100°C for five min using flame. Formation of bluish green color showed the presence of anthocyanin and formation of yellow color indicated the presence of betacyanins. Carbohydrates were identified as per reported procedure. About 0.2 g of each plant extract/fractions were dissolved in distilled water and then few drops of Molisch's reagent were added, followed by addition of 1 mL conc. H_2_SO_4_. After 2 minutes, the mixture was diluted with 5 mL distilled water. Formation of a red or dull violet color at the interphase of two layers was considered as a positive test. The total phenolic contents of 80% MeOH and fractions of* Hypochaeis radecata *were determined using Folin-Ciocalteu reagent according to the method described by [6]. Gallic acid was used as standard. In this method, 100 *μ*l of each extract or fractions in concentrate of 100 *μ*g/ml were combined with 500 *μ*l of the Folin-Ciocalteu reagent and 1.5 ml of sodium carbonate (20%). The mixture was shaken and made up to 10 ml using distilled water and allowed to stand for 2 hrs. Then the absorbance was measured at 765 nm. All determinations were carried out thrice. The total phenolic content was calculated as gallic acid equivalent (GAE) by the following equation: *T* = CxV/M. *T* is the total phenolic content in mg·g^–1^ of the extracts as GAE, *C* is the concentration of gallic acid established from the calibration curve in mg·ml^–1^, *V* is the volume of the extract solution in ml, and *M* is the weight of the extract in *g*.

### 2.4. Molecular Docking

The molecular docking study of isolated phytochemicals, compounds** 1** and** 2**, from* H. radicata* (shown in Figure 1), were carried out to recognize the molecular interaction of the phytochemicals (**1** and** 2**) within the active site of cyclooxygenases COX-1 (PDB ID 4O1Z) and COX-2 (PDB ID 5F19) by captivating X-rays crystal structure data of enzymes from protein data bank (PDB) [7]. Molecular docking analysis essentially involves the selection and grounding of suitable protein and ligand, following by the screening of docking output and their interaction. Both ligands and the receptors were prepared distinctly. The protein structure of both COX-1 and COX-2 was independently preprocessed using structure preparation wizard [7], and omitted hydrogen was also added. The hydrogen bonds inside the proteins were also optimized and monitored by the minimization of protein with default setup of MOE (Molecular Operating Environment). The unsolicited water molecules and cofactors were detached from the proteins. The chain A was designated in the “refine” tab. The molecular docking used in this study predicts the binding modes, biding energy, binding affinity, and orientation of ligands at active site of COX-1 and COX-2.

### 2.5. *In Vivo* Biological Assays

#### 2.5.1. Animals

BALB/C mice were used in this* in vivo* screening. The mice used in this finding were fed with standard laboratory food and also with water ad libitum. All the animals used in this study were kept under standard conditions of temperature and light before starting the experiments. The animals were adjusted to the laboratory conditions. The experimental procedures were approved by the Ethical Committee (UOP/Pharm-6) of the Pharmacy Department, University of Peshawar, Peshawar, Pakistan. For animal testing, the animals were divided into 14 groups for each activity (Table 1): two groups were normal control and reference group and the remaining groups were treated with different concentration of crude extract or fractions. The treatments were administered intraperitoneally (i.p.) and extracts were dissolved or suspended in distilled water.

#### 2.5.2. Acute Toxicity Assay

The animals were divided into 14 groups; each group has six animals (*n* = 6). The crude extracts and the various fractions of* H. radicata *samples at 250, 500, and 1000 mg/kg (body weight of mice) were used as test dose (Table 1). After intraperitoneally (i.p.) administering the test doses, the animals were kept under observation for 24 hours, for any behavioral effects and mortality. The numbers of survived and dead animals were noted by calculating the percentage of mortality [8].

#### 2.5.3. Acetic Acid-Induced Writhing Test

The analgesic activity of crude extracts and the fractions was investigated by using the acetic acid-induced abdominal constriction test. The animals were divided into various groups (Table 1); each group has six animals (*n* = 6), weighing 18–22 g. One group received normal saline (10 ml/kg, i.p.) as control, and the second group received diclofenac (10 mg/kg, i.p.) as a standard analgesic drug. The remaining groups received extracts and fractions at the doses of 25, 50, and 100 mg/kg (i.p.). After 40 minutes of the treatment, pain was induced by intraperitoneal injection of 0.9% acetic acid (v/v, 0.1 ml/10 g body weight). The number of muscular contractions was counted over a period of 20 min after acetic acid injection. The number of writhes in each treated group was compared with control (saline-treated group) [9]:

(1) $$\begin{array}{c} \%\;\;protection=\frac{no.\;\;of\;\;writhing\;\;in\;\;control\;\;-\;\;no.\;\;of\;\;writhing\;\;in\;\;test\;\;compound}{no.\;\;of\;\;writhing\;\;in\;\;control}\times 100. \end{array}$$

#### 2.5.4. Muscle-Relaxant Activity

*(1) Inclined Plan Test*. The fractions of* H. radicata *extract were evaluated in inclined-plane model [10]. The plane used in this procedure consisted of two plywood boards. Both the boards were connected with each other in such a way that one board formed the base and the other is fixed to the base at 65 degrees. Different groups were treated with diazepam (1 mg/kg), normal saline (10 ml/kg), and the test fractions (Table 1). After administration with interval of 30, 60, and 90 minutes, the animals were placed on the upper part of the inclined plane for 30 seconds to hang or fall.

*(2) Traction Test. *In this technique, a metal wire coated rubber was used; both ends of the wire were rigidly stretched and supported with stands, about 60 cm above a laboratory bench. The animals were clustered into 14 groups (*n* = 6) as shown in Table 1. The two groups were treated with normal saline (10 ml/kg) and diazepam (1 mg/kg) as a positive and negative control group, respectively. The rest of the groups were treated with crude extract, n-hexane, chloroform, and ethyl acetate fractions of the crude extract of* H. radicata*, in correspondent doses of 25, 50, and 100 mg/kg of body weight. The traction test was performed for all the treated animals, with time interval of 30, 60, and 90 minutes, after the administration of treatment. Each animal was suspended from hind legs on the wire and the time of floppy was recorded for 5 s. The failure to hang in less than five seconds reflects the presence of muscle-relaxant property and vice versa [8].

#### 2.5.5. Sedative Activity

Open field test is used to investigate the sedative potential of a drug. The apparatus design for sedative potential consists of an area of white wood (150-cm diameter), enclosed by stainless steel walls and divided in 19 squares by black lines. The open field was placed in a light and sound-attenuated room. BALB/c mice of either sex (*n* = 6), weighing 26 ± 4 g, were used in this study. The animals were acclimatized under red light (40-Watt red bulb) one hour before the commencement of the experiment, with food and water available ad libitum. The animals were administered with normal saline as a negative control (10 ml/kg) and diazepam as a reference drug (0.5 g/kg). The rest of the groups were treated with the crude extract and the fractions of* H. radicata* in serial doses of 25, 50, and 100 mg/kg. After 30 min, each animal was placed in the center of the box and the number of lines crossed was counted for each mouse, following related literature [11, 12].

### 2.6. Statistical Analysis

Results are expressed as mean ± SEM. One-way ANOVA (analysis of variance) was used for a comparison test of significant differences among groups, followed by Dunnett's multiple comparison posttest. A level of significance (*P* < 0.05 or 0.01) was considered for each test. The results of activities were also presented as mean ± SEM, and their statistical analysis was carried out using the GraphPad program (GraphPad, San Diego, CA, USA).

## 3. Results

### 3.1. Phytochemical Composition

The results of the phytochemical composition of crude extracts, as well as the polar and nonpolar organic fractions, are given in Tables 2(a) and 2(b). The phytochemical screening test exhibited the presence of alkaloids, flavonoids, glycosides, terpenoids, phlobatannins, tannins, reducing sugars, saponins, steroids, and total phenolic. Luteolin or isoetin is likely to be the flavonoid, as discovered in other species of* Hypochaeris* [11].

The chloroform fractions contained alkaloids, tannins, glycosides, steroids, and terpenoids. Among other fractions, ethyl acetate showed the presence of maximum number of phytochemicals including alkaloids, tannins, glycosides, reducing sugars, saponin, flavonoids, phlobatannins, steroids, and terpenoids, while hexane showed only nonpolar phytochemicals such as steroids and terpenoids. The phytochemicals play the key role in the bioactivity of extract.

### 3.2. Molecular Docking Analysis

**Figure 2:**
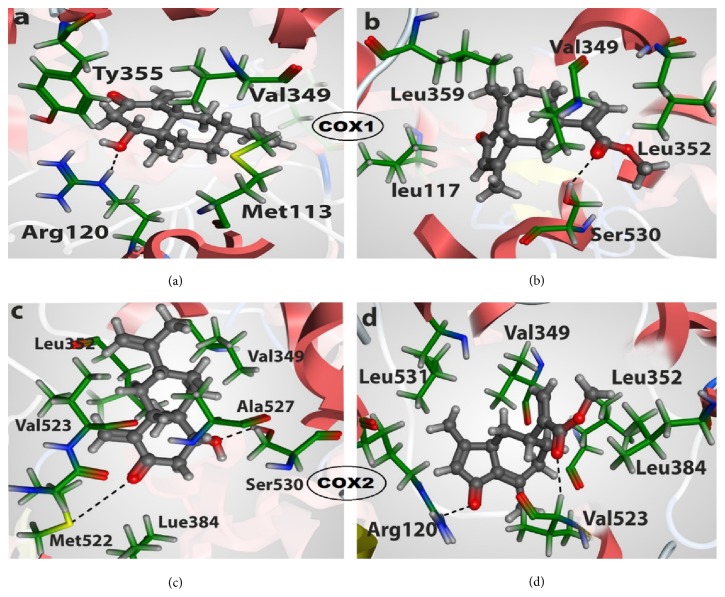
3D representation of compound** 1** and** 2** (gray) interactions (black) with the active sites amino acids (green) of COX1 and COX2 enzyme. (a) Docked pose of compound** 1** in the active site of COX1. (b) Docking interactions of compound** 2** with the active site key amino acids of COX1. (c) Interactions of compound** 1** with COX2 active site residues. (d) Docked pose of compound** 2** with the important active site residues of COX2 enzyme.

Molecular docking studies were performed with the help of MOE software, to study the binding interactions involved between the active sites of protein and the selected compounds. The compounds** 1** and** 2** (Figure 1) were docked in the active site of COX-1 enzyme (Figure 2), and the hydrogen bond and few hydrophobic interactions were observed with the important active site amino acids. Compound** 1** had common pharmacophoric scaffold. The hydroxyl group made proper hydrogen bond with Arg120 and also exhibited some hydrophobic interactions with Met113, Val49, and Tyr355 (Figure 2(a)), having good docking score and binding affinity (Table 3). The carbonyl oxygen of compound** 2** forms single hydrogen bond with the key residue Ser530, whereas few hydrophobic interactions with Lue117, Val349, Leu352, and Leu359 were observed (Figure 2(b)).

In case of COX-2 enzyme, the docking studies revealed two hydrogen bonds and few hydrophobic interactions with crucial active site amino acids with compounds** 1** and** 2**, respectively. Compound** 1** showed two hydrogen bonds towards Met522 and Ser530, while hydrophobic interactions were observed with Val523, Ala527, Val349, and Leu352 (Figure 2(c)). Compound** 2 **also showed two hydrogen bonds with the Arg120 and Val523. The hydrophobic interactions were shown by Val349, Leu352, Leu531, and Leu384 active site residues. The docking results revealed good docking score, good binding affinities (Table 3), and important interactions of the compounds** 1** and** 2** with the key residues of active sites of COX1 and COX-2 enzymes.

### 3.3. In Vivo Biological Effects

#### 3.3.1. Acute Toxicity Study

The acute toxicity of combine crude extract and different fractions of* H. radicata* was assessed in various doses (250, 500, and 100 mg/kg; Table 4). During 24 hr assessment of the important behavioral of toxicity, no mortality was observed at higher doses, though the animals showed slight sedation after 1 hr of the administration of crude extracts and various isolated fractions. The safe treatment doses range 25–100 mg/kg of extract and its fractions is selected for further treatment and studies.

#### 3.3.2. Analgesic Activity

**Figure 3:**
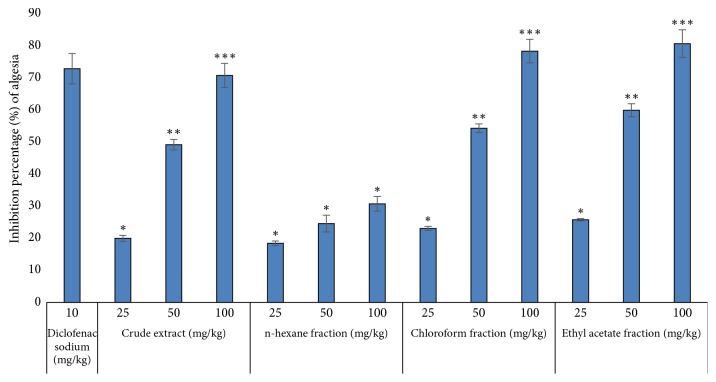
Percentage of inhibition of algesia by extract and fractions of* H. radicata*. The values are percentage ± SEM for animals (*n* = 6). ^*∗*^*P* < 0.05, ^*∗∗*^*P* < 0.01, ^*∗∗∗*^*P* < 0.001, all compared with control.

The acetic acid-induced writhing was evidently reduced by the crude extract/fractions of* H. radicata*, as presented in Table 5 and Figure 3. The crude extract of the plant significantly inhibited the writhing dose dependently, with inhibition percentage values of 20% (*P* < 0.05), 49.23% (*P* < 0.05), and 70.84% (*P* < 0.001) at the doses of 50, 100, and 150 mg/kg, respectively. The chloroform and ethyl acetate fractions of the plant have shown a significant analgesic activity in dose-dependent manner with the maximum inhibition values of 78.46% and 80.76% at dose of 100 mg/kg, respectively. The n-hexane fraction exhibited very mild analgesic activity, that is, 18, 25, and 30 percent inhibition at doses of 25, 50, and 100 mg/kg, respectively.

#### 3.3.3. Muscle-Relaxant Effect

The effects of extract/fractions of* H. radicata *at 25, 50, and 100 mg/Kg i.p. in inclined-plane test and traction test are given in Table 6. The percentage of muscle-relaxant activity is presented for extracts/fractions at intervals of 30, 60, and 90 minutes after administration of the extract/fractions. The activity was compared with that of reference drug diazepam, efficacy of which is considered to be 100%. The crude extract and fraction exhibited muscle-relaxant activity in dose-dependent manner, compared to saline-treated group; however, it was observed to be very mild in comparison to diazepam. The maximum muscle-relaxant activity was observed after 90 minutes at a dose of 100 mg/kg of each fraction; however, it was relatively less in the n-hexane fraction. It is noteworthy that the effect is mild but tends to be dose-dependent.

#### 3.3.4. Sedative Activity

**Figure 4:**
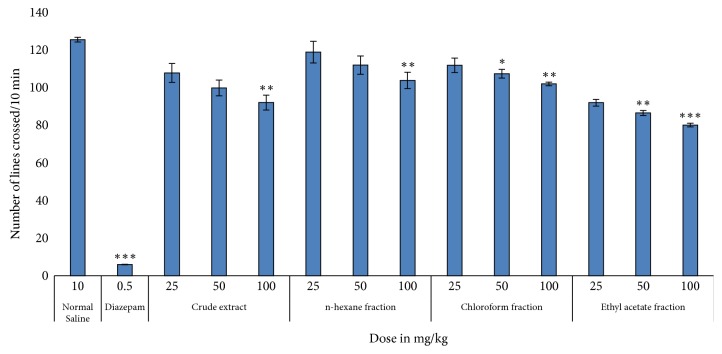
Sedative activity of crude extract and its fractions of* H. radicata.* The values represent locomotion ± SEM (*n* = 6). ^*∗*^*P* < 0.05, ^*∗∗*^*P* < 0.01, ^*∗∗∗*^*P* < 0.001, all compared with control.

The locomotive potency in mice at test doses of crude extract and its fractions of* H. radicata *is depicted in Table 7 and Figure 4. Our results showed that crude extract and its fractions exhibited significant sedative effect at 25, 50, and 100 mg/kg i.p., as compared to the saline-treated group. However, n-hexane fraction exerted relatively less sedative effect in minimum dose (i.e., 25 mg/kg). The prominent sedative effect was observed in ethyl acetate fraction. However, in all the fractions, the effect is very mild, compared to diazepam-treated group.

## 4. Discussion

*H. radicata *has wide folkloric medicinal uses [1–3], but the experimental verification of these effects is scanty. The isolated extract/fractions were assessed for the presence of various phytochemical groups, by using conventional chemical procedures. However, the quantitation of phytochemicals is not achieved yet. The ethyl acetate fraction was found to have almost all the phytochemicals found in the crude extract, and it is regarded as chemically rich fraction of* H. radicata*. During literature survey about the plant, we found that four various compounds have been isolated from* H. radicata*, out of which two molecules are volatile in nature, and the other two molecules are compounds** 1** and** 2**, respectively [2]. We suggested that analgesic and anti-inflammatory properties of* H. radicata* might be partly due to these nonvolatile compounds. Preliminarily the two compounds were subjected to docking analysis for the inhibition of COX-1 and COX-2. Our data (Figure 1 and Table 3) revealed good docking score, good binding affinities (Table 3), and important interactions of the compounds** 1** and** 2** with the key residues of active sites of COX-1 and COX-2 enzymes. However, the predominant interaction was observed between COX-1 and compound** 1**. This enzyme is responsible for the production of the lipid autacoids such as the prostaglandins, which mediate inflammatory response [12]. However, this is preliminary study of compounds** 1** and** 2**, and it will be verified by* in vitro* and* in vivo* assays in the future.

The safe dose range for the administration of the extracts/fractions of* H. radicata* in the animals was determined by toxicity studies, and no evident toxicity or mortality was observed in far higher doses (1000 mg/kg). For the* in vivo* studies, 5–100 mg/kg dosage was tested. The analgesic activity of the crude extract and it fractions was carried out in acetic acid-induced writhing model, which is well recommended model in in estimating medicinal agents for their analgesic potency [13, 14]. The feeling of pain is brought by liberation of endogenous constituents including prostaglandin and arachidonic acid via the cyclooxygenases (COX) [7, 9]. The local peritoneal receptors could be the origin of abdominal writhing [14]. The pain sensation in acetic acid-induced writhing model is provoked by making localized inflammatory response due to the release of free arachidonic acid from tissue phospholipids through phospholipases and the creation of prostaglandins, specifically PGE2 and PGF2. The level of lipoxygenase products could also rise in peritoneal fluids [7]. These prostaglandin and lipoxygenase products cause inflammation and pain by growing the capillary permeability. The constituents inhibiting the writhing will have analgesic potency by the inhibition of prostaglandin synthesis [9]. In our study, significant analgesic effect of the crude extract, chloroform, and ethyl acetate fractions of* H. radicata* was observed. This led to the assumption that the analgesic phytochemical constituents of this studied plant are polar molecules.

The sedative potency of the title plant was evaluated by applying the open field test (locomotive activity). Open fields screening is normally employed as a prognostic test for the calculation of sedative activity [11, 12]. Pretreatment of mice with crude extract and various fractions exhibited dose-dependent reduction in locomotive properties in the open field test, associated with control, but this decline was far less than that produced in diazepam-treated animals. The reduction in the frequency and amplitude of motion could be attributed to the sedative effect of* H. radicata*, which was found to exert mild sedation compared to diazepam.

The inclined-plane test and traction test were performed in animals for determining the muscle-relaxant potency of the treatments [8]. The animals used in this model are permitted to spend time on the rotating rod, where less time spent on the rod designates a muscle-relaxant effect of a tested material. Results from this study revealed that all fractions of* H. radicata *extract/fractions had mild muscle-relaxant activity. This mild effect was observed to be dose-dependent.

Researchers thought that the sedative and muscle-relaxant-like properties of benzodiazepines such as bromazepam are frequently due to interference with the action of gamma aminobutyric acid (GABA) [15]. Furthermore, finding indicated that benzodiazepines are fixed to the gamma subunit of the GABA_A_ receptor, thus enhancing the affinity GABA receptor for *γ*-amino butyric acid (GABA). GABA binding to its receptor increases chloride ion conductance and inhibition of the action potential. The whole effects of crude extract and its isolated fractions of* H. radicata *were similar to standard drug used (diazepam).

Some other Asteraceae family plants with medicinal values include* Artemisia scoparia* (redstem wormwood) [16]. Other members of this family that have been studied for their medicinal value include wormwood, Israel's chamomile, tridax daisy, Chinese Wedelia, sweetleaf, mugwort, thistle, and meadow fleabane. Marsh thistle (*Cirsium palustre* and* Cirsium rivulare*) root essential oil exerted antiproliferative activity against MCF7 and MDA-MBA-231 breast cancer cell lines due to their constituent [17].

Some other Asteraceae family members also inhibit T cell activation, MAPK phosphorylation, and NF-*κ*B activities [18].

Though the results appear promising, assessing the risks of a pharmaceutical candidate is important.* H. radicata* is known to be a neurotoxic plant for some herbivore animals, via* inducing* motor hyperactivity [19]. This plant elaborates sesquiterpenes (germacrene-, eudesmane-, and guaiane-type). Sesquiterpenes are known to cause human health issues. The Asteraceae family member* Parthenium hysterophorus* is known to cause hypersensitivity in herbivore as well as humans [19].

## 5. Conclusion

The extract/fractions of* H. radicata *showed significant analgesic effect in the* in vivo* model of peripheral algesia. The docking analysis of previously isolated molecules from the plant also exhibited promising interactions with the inflammatory enzymes COX-1 and COX-2. The plant has a mild sedative and muscle-relaxant potential. Thus, this study justified the rationale for the traditional use and prospected pharmacological scopes of this plant as analgesic and anti-inflammatory remedy.

## Figures and Tables

**Table 1:** The treatment groups in each animal test. Each treatment group contains 6 animals (*n* = 6). All treatments were administered intraperitoneally (i.p.).

Treatment groups	Toxicity testing	Acetic acid induced writhing test	*Inclined plan test/traction test*	Sedative activity
Normal Saline	10 ml/kg	10 ml/kg	10 ml/kg	10 ml/kg

Reference group	-* *-* *-* *-* *-	Diclofenac (10 mg/kg)	Diazepam (1 mg/kg)	Diazepam (0.5 mg/kg)

Crude extract	250 mg/kg	25 mg/kg	25 mg/kg	25 mg/kg
	500 mg/kg	50 mg/kg	50 mg/kg	50 mg/kg
	1000 mg/kg	100 mg/kg	100 mg/kg	100 mg/kg

n-Hexane fraction	250 mg/kg	25 mg/kg	25 mg/kg	25 mg/kg
	500 mg/kg	50 mg/kg	50 mg/kg	50 mg/kg
	1000 mg/kg	100 mg/kg	100 mg/kg	100 mg/kg

Chloroform fraction	250 mg/kg	25 mg/kg	25 mg/kg	25 mg/kg
	500 mg/kg	50 mg/kg	50 mg/kg	50 mg/kg
	1000 mg/kg	100 mg/kg	100 mg/kg	100 mg/kg

Ethyl acetate fraction	250 mg/kg	25 mg/kg	25 mg/kg	25 mg/kg
	500 mg/kg	50 mg/kg	50 mg/kg	50 mg/kg
	1000 mg/kg	100 mg/kg	100 mg/kg	100 mg/kg

**Table:** (a) Phytochemical composition of crude extract and various fractions of *H. radicata, *where the signs (+) and (−) represent the presence and absence, respectively.

Phytochemicals	Crude extract	n-Hexane fraction	Chloroform fraction	Ethyl acetate fraction
Alkaloids	**+**	**−**	**+**	**+**
Anthraquinones	**−**	**−**	**−**	**+**
Tannins	**+**	**−**	**+**	**+**
Glycosides	**+**	**+**	**+**	**+**
Reducing sugars	**+**	**−**	**−**	**+**
Saponins	**+**	**−**	**−**	**+**
Flavonoids	**+**	**−**	**−**	**+**
Phlobatannins	**+**	**−**	**−**	**+**
Steroids	**+**	**+**	**+**	**+**
Terpenoids	**+**	**+**	**+**	**+**
Coumarin	**−**	**−**	**−**	**−**
Emodin	**−**	**−**	**−**	**−**
Anthocyanin	**−**	**−**	**−**	**−**

**Table:** (b) Identification of total phenolic contents of crude extract and various fractions of *H. radicata*

Phytochemicals	Crude extract	n-Hexane fraction	Chloroform fraction	Ethyl acetate fraction
Total phenolic contents (GAE)	19.17	9.36	8.15	11.73
SD	2.85	1.57	2.48	2.08

**Table 3:** Showing the docking scores, binding energies, and binding affinities of the compounds with the active sites of the COX1 and COX2.

S. no.	COX-1			COX-2		
	Docking score	Binding energy (Kcal/mol)	Binding affinity (Kcal/mol)	Docking score	Binding energy (Kcal/mol)	Binding affinity (Kcal/mol)
Compound **1**	−7.547	−34.25	−39.32	−6.609	−35.79	−41.99
Compound **2**	−8.341	−7.03	−7.55	−6.887	−7.37	−7.75

**Table 4:** Acute toxicity of crude extract and its fractions of *H. radicata*.

Treatment	Dose (mg/kg)	No. of animals died/5	% mortality
Normal saline	10 ml/kg	0/5	0

Crude extract	250	0/5	0
	500	0/5	0
	1000	0/5	0

n-Hexane fraction	250	0/5	0
	500	0/5	0
	1000	0/5	0

Chloroform fraction	250	0/5	0
	500	0/5	0
	1000	0/5	0

Ethyl acetate fraction	250	0/5	0
	500	0/5	0
	1000	0/5	0

**Table 5:** *Analgesic potential of crude extract and its fractions of H. radicata*. Values represent the number of writhing, 30 minutes after treatment with distilled water (10 ml/kg, control), crude methanolic extract, and its various fractions (25, 50, and 100 mg/Kg), or diclofenac (10 mg/kg). Data presented as mean ± SEM (*n* = 6). ^*∗*^*P* < 0.05, ^*∗∗*^*P* < 0.01, ^*∗∗∗*^*P* < 0.001, all compared with control.

Treatment	Dose	No. of writhing ± SEM
Normal saline	10 ml/kg	65 ± 3.46

Diclofenac sodium	10 mg/kg	17.6 ± 1.14

Crude extract	25 mg/kg	52 ± 2.44^*∗*^
	50 mg/kg	33 ± 1.10^*∗∗*^
	100 mg/kg	17 ± 0.87^*∗∗*^

n-Hexane fraction	25 mg/kg	53 ± 2.11^*∗*^
	50 mg/kg	49 ± 1.77^*∗*^
	100 mg/kg	45 ± 0.48^*∗*^

Chloroform fraction	25 mg/kg	50 ± 1.44^*∗*^
	50 mg/kg	29.67 ± 0.76^*∗∗*^
	100 mg/kg	14 ± 0.66^*∗∗∗*^

Ethyl acetate fraction	25 mg/kg	48.23 ± 0.68^*∗*^
	50 mg/kg	26. ± 0.88^*∗∗*^
	100 mg/kg	12.50 ± 67^*∗∗∗*^

**Table 6:** Muscle relaxant activity of crude extract and its fractions of *Hypochaeris radicata*. The values are presented as percentage ± SEM for each animal (*n* = 6).

Groups	Dose (mg/kg)	Inclined plan test (%)			Traction test (%)		
		30 min	60 min	90 min	30 min	60 min	90 min
Distilled water	10 ml	0 ± 0.00	0 ± 0.00	0 ± 0.00	0 ± 0.00	0 ± 0.00	0 ± 0.00

Diazepam	1	100 ± 0.00	100 ± 0.00	100 ± 0.00	100 ± 0.00	100 ± 0.00	100 ± 0.00

Crude extract	25	1.44 ± 0.56	2.55 ± 0.77	3.21 ± 0.55	1.48 ± 0.56	2.53 ± 0.64	4.31 ± 0.50
	50	1.87 ± 0.57	4.18 ± 0.69	4.20 ± 1.00	1.84 ± 0.60	3.56 ± 0.69	5.10 ± 0.70
	100	3.50 ± 0.58	4.97 ± 0.88	7.00 ± 1.18	3.65 ± 0.63	4.70 ± 0.60	7.01 ± 1.14

n-Hexane fraction	25	1.25 ± 0.50	3.00 ± 0.55	3.24 ± 0.66	1.30 ± 0.56	3.25 ± 0.55	3.78 ± 0.60
	50	2.57 ± 0.70	4.10 ± 0.97	4.33 ± 0.88	2.68 ± 0.73	4.27 ± 0.95	4.76 ± 0.99
	100	4.30 ± 0.59	4.12 ± 0.77	6.00 ± 1.00	4.05 ± 0.59	4.25 ± 0.70	6.63 ± 1.00

Chloroform fraction	25	1.66 ± 0.52	2.77 ± 0.78	3.82 ± 0.66	1.68 ± 0.87	2.66 ± 0.66	4.55 ± 0.52
	50	1.95 ± 0.65	4.88 ± 0.80	4.50 ± 0.60	2.04 ± 0.84	3.80 ± .87	5.44 ± 0.60
	100	3.66 ± 0.78	5.20 ± 0.59	7.60 ± 0.90	3.90 ± 0.60	4.97 ± 0.80	7.50 ± 1.10

Ethyl acetate fraction	25	1.88 ± 0.60	2.90 ± 0.63	3.98 ± 0.68	2.00 ± 0.88	3.01 ± 0.60	5.50 ± 0.63
	50	2.20 ± 0.63	3.30 ± 0.60	4.50 ± 0.77	2.98 ± 0.76	4.15 ± 0.65	6.45 ± 0.60
	100	4.63 ± 0.70	5.20 ± 0.77	6.00 ± 0.88	4.40 ± 0.64	5.98 ± 0.65	7.80 ± 0.80

**Table 7:** *Sedative activity of crude extract and its fractions of H. radicata*. Values represent the number of lines crossed by an animal in a box, 30 minutes after treatment with distilled water (10 ml/kg, control), crude methanolic extract, and its various fractions (25, 50, and 100 mg/Kg), or diazepam (0.5 mg/kg). Data presented as mean ± SEM (*n* = 6). ^*∗*^*P* < 0.05, ^*∗∗*^*P* < 0.01, ^*∗∗∗*^*P* < 0.001, all compared with control.

Sample	Dose	No. of lines crossed/10 min
Distilled water	10 ml/Kg	126 ± 1.25

Diazepam	0.5 mg	6 ± 0.13^*∗∗∗*^

Crude extract	25 mg/kg	108.25 ± 5.01
	50 mg/kg	100.24 ± 4.22
	100 mg/kg	92.45 ± 4.00^*∗∗*^

n-Hexane fraction	25 mg/kg	119.48 ± 5.80
	50 mg/kg	112.44 ± 4.87
	100 mg/kg	104.25 ± 4.35^*∗∗*^

Chloroform fraction	25 mg/kg	112.35 ± 3.88
	50 mg/kg	107.85 ± 2.33^*∗*^
	100 mg/kg	102.39 ± 1.00^*∗∗*^

Ethyl acetate fraction	25 mg/kg	92.35 ± 1.77^*∗∗∗*^
	50 mg/kg	86.88 ± 1.33^*∗∗*^
	100 mg/kg	80.41 ± 1.00^*∗∗∗*^

## Supplementary Materials

Supplementary Materials Graphical abstract of this paper is given as a supplementary material. Supplementary material represents in vivo study on analgesic, muscle-relaxant, sedative activity of extracts of* Hypochaeris radicata*.
