# Supplementary material for: In Vivo Study on Analgesic, Muscle-Relaxant, Sedative Activity of Extracts of Hypochaeris radicata and In Silico Evaluation of Certain Compounds Present in This Species
Source: Biomed Res Int. 2018 May 13;2018:3868070. doi: 10.1155/2018/3868070 (PMC6016213; doi:10.1155/2018/3868070)
Supplement: Supplementary Materials — Graphical abstract of this paper is given as a supplementary material. Supplementary material represents in vivo study on analgesic, muscle-relaxant, sedative activity of extracts of Hypochaeris radicata. [file 3868070.f1.docx]

Supplementary material represent in vivo study on analgesic, muscle-relaxant, sedative activity of extracts of *Hypochaeris radicata*
